# Right basal bronchial fistula due to amebic infection: a case report

**DOI:** 10.1186/s12890-023-02412-9

**Published:** 2023-04-14

**Authors:** Tomohiro Yazawa, Hitoshi Igai, Mitsuhiro Kamiyoshihara, Ken Shirabe

**Affiliations:** 1 Division of General Thoracic Surgery, Integrative Center of General Surgery, Gunma University Hospital, 3-39-22, Showa-Machi, Maebashi, 371-8511 Gunma Japan; 2 Department of General Thoracic Surgery, Japanese Red Cross Maebashi Hospital, Maebashi, Gunma Japan

**Keywords:** Bronchial fistula, Ameba, HIV, Liver abscess, Surgery

## Abstract

**Background:**

Pleuropulmonary amebiasis is the second most common form of extraintestinal invasive amebiasis, but cases that include bronchopleural fistula are rare.

**Case presentation:**

A 43-year-old male was referred to our hospital for liver abscess, right pleural effusion, and body weight loss. He was diagnosed with a bronchopleural fistula caused by invasive pleuropulmonary amebiasis and human immunodeficiency virus (HIV) infection. After initial medical treatment for HIV infection and invasive amebiasis, he underwent pulmonary resection of the invaded lobe. Intraoperative inspection revealed a fistula of the right basal bronchus in the perforated lung abscess cavity, but the diaphragm was intact. The patient was discharged on postoperative day 3 and was in good condition at the 1-year follow-up.

**Conclusions:**

Clinicians should be aware that pleuropulmonary amebiasis can cause a bronchopleural fistula although it is very rare.

## Background

Pleuropulmonary amebiasis is the second most common form of invasive extraintestinal amebiasis, and is frequently accompanied by amebic liver abscesses [1]. While invasive amebiasis is often accompanied by pleural empyema, this is the first report of its causing bronchopleural fistula. The patient was successfully treated by surgical resection of the invaded pulmonary lobe.

## Case presentation

**Fig. 1:**
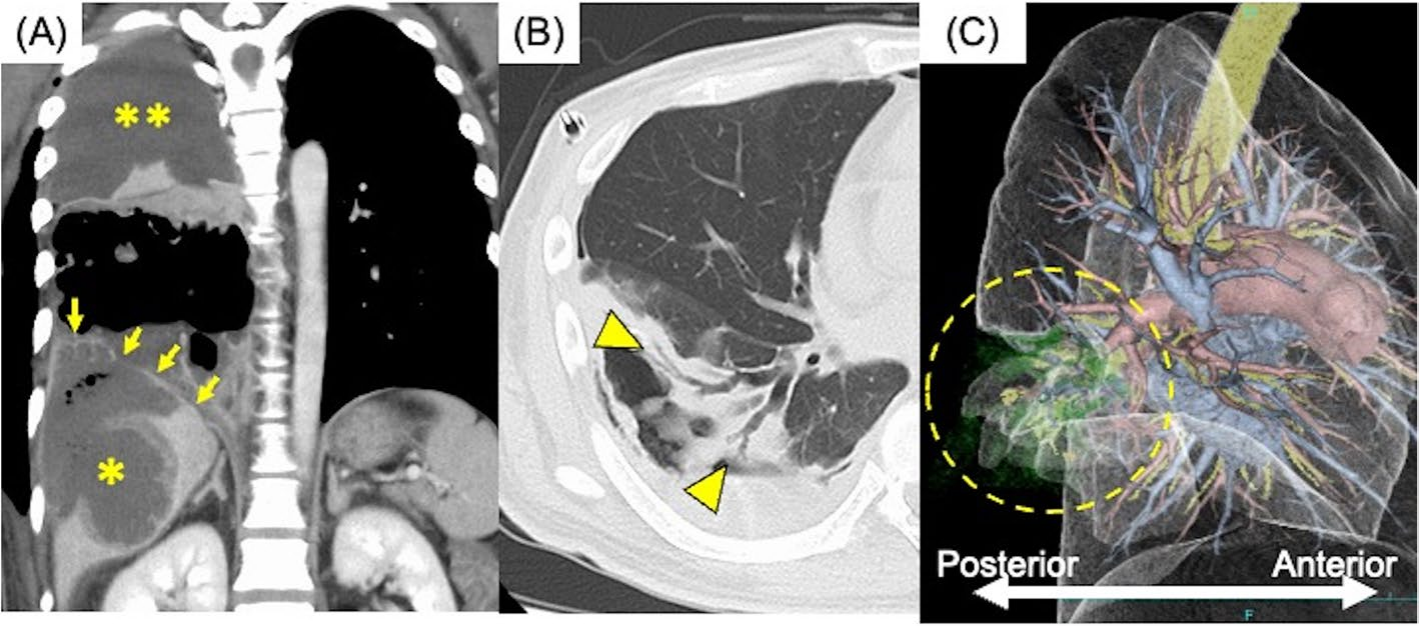
Computed tomography (CT) showed (**A**) an amebic abscess of the liver (yellow single asterisk) and amebic pleuropulmonary empyema (yellow double asterisk), although the diaphragm was intact (yellow arrows). **B** A bronchial fistula was identified in the right basal bronchus (yellow arrowheads). **C** Three-dimensional CT revealed a broad defect of the right lower lobe due to amebic infection (yellow dotted circle)

**Fig. 2:**
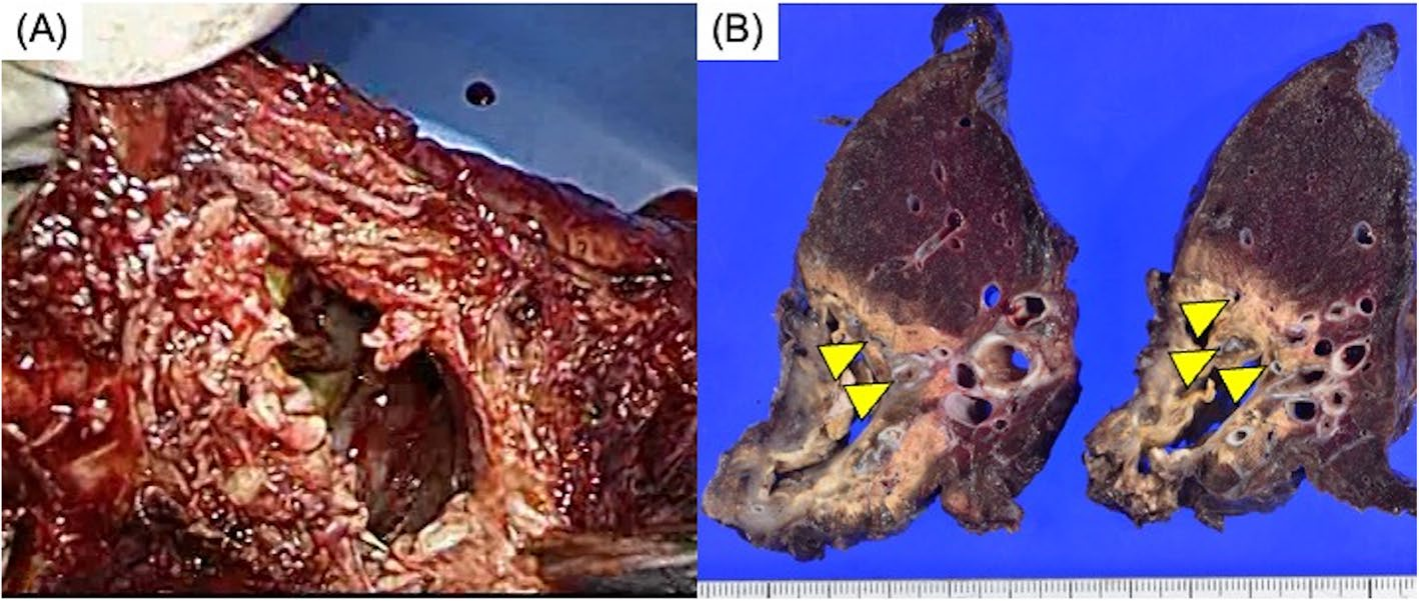
**A** Intraoperative examination revealed an extensive defect in the posterior basal segment of the right lower lobe, consistent with a perforated lung abscess cavity. **B** Pathological findings revealed multiple bronchial fistulas of the right lower lobe in the perforated lung abscess cavity (yellow arrowheads)

A 43-year-old male was referred to our hospital for liver abscess, right pleural effusion, and body weight loss (31 kg within 6 months). He had no past medical history. Laboratory tests revealed human immunodeficiency virus (HIV) and amebic infection (both untreated) using real-time PCR and western blot. Because the patient had dyspnea and his blood gases showed desaturation, we inserted a thoracostomy tube to reduce the massive pleural effusion. The pleural effusion was anchovy paste-like and we detected *Entamoeba histolytica*, which was also found in his stool. Although the patient had persistent air leakage, he did not cough up pus. The liver abscess was treated conservatively. HIV treatment was initiated, and the amebiasis was treated concurrently with metronidazole followed by paromomycin (10 days, respectively). While the pleural fluids subsequently became serous, the air leakage persisted. Computed tomography (CT) suggested a bronchopleural fistula of the right basal bronchus (Fig. 1), whereas the liver abscess was no longer visible. Roughly 2 months after HIV treatment initiation, his HIV-RNA copy number had decreased and was within the safe range. His general condition was improved dramatically, and his body weight increased to 69.5 kg. He then underwent open right lower lobectomy, during which a fistula between the bronchus and lung abscess was detected (Fig. 2A). The diaphragm was intact, and there was no sign of a ruptured liver abscess involving the thoracic cavity. The duration of surgery was 150 min, and the blood loss was 100 ml. Pathological findings revealed a right basal bronchial fistula in the perforated lung abscess (Fig. 2B). The chest drainage tube was removed on postoperative day 2. The patient was discharged on postoperative day 3 and was in good condition at the 1-year follow-up.

## Discussion and conclusions

Amebic infection with *Entamoeba histolytica* occurs worldwide but is most common in subtropical and tropical countries [2]. Low socioeconomic status, malnutrition and chronic alcoholism contribute to the development of amoebiasis. Amebic liver abscess is the most common extraintestinal complication, followed by pleuropulmonary complications, which are often associated with liver abscesses [1]. Although the pathogenesis of empyema due to amebic infection is often rupture of a liver abscess into the thoracic cavity via the diaphragm, amebic empyema developing from a lung abscess has also been reported. To the best of our knowledge, this is the first report of a bronchial fistula due to amebic infection that occurred independently from a liver abscess. In this case, i) the diaphragm (as seen on CT) was intact although the liver abscess had ruptured into the abdominal cavity (Fig. 1A); ii) amoebae were confirmed in his stool; and iii) the diaphragm was also intact intraoperatively. These findings suggested that the bronchial fistula had developed from the lung abscess due to hematogenous amebic infection from the intestine.

There were two preoperative issues in this case. First, the patient’s initial condition did not allow surgery due to HIV/AIDS. It has been reported that the HIV-positive patient who had the number of HIV-RNA copies more than 3 × 10^4^ copies/ml has a high complication risk (his HIV viral load was 7.1 × 10^5^ copies/ml) [3]. Second, his HIV-RNA copy number was sufficiently elevated to pose a high risk of the HIV transmission to healthcare workers [4–6]. Therefore, the HIV and amebic infections were treated first, and concurrently, before surgery was performed.

In conclusion, we present a case in which a bronchopleural fistula was caused by invasive amebiasis. The patient recovered after surgical resection of the invaded pulmonary lobe. Clinicians should be aware that, although very rare, pleuropulmonary amebiasis can cause bronchopleural fistula.

## Data Availability

All data and material are available for sharing if needed.

## Abbreviations

CT: Computed tomography
HIV: Human immunodeficiency virus
